# Time-Dependent Vascular Effects of Endocannabinoids Mediated by Peroxisome Proliferator-Activated Receptor Gamma (PPAR*γ*)

**DOI:** 10.1155/2009/425289

**Published:** 2009-04-29

**Authors:** Saoirse E. O'Sullivan, David A. Kendall, Michael D. Randall

**Affiliations:** ^1^School of Graduate Entry Medicine and Health, Derby City General Hospital, University of Nottingham, Derby DE22 3DT, UK; ^2^School of Biomedical Sciences, Queen's Medical Centre, University of Nottingham, Nottingham NG7 2UH, UK

## Abstract

The aim of the present study was to examine whether endocannabinoids cause PPAR*γ*-mediated vascular actions. Functional vascular studies were carried out in rat aortae. Anandamide and N-arachidonoyl-dopamine (NADA), but not palmitoylethanolamide, caused significant vasorelaxation over time (2 hours). Vasorelaxation to NADA, but not anandamide, was inhibited by CB_1_ receptor antagonism (AM251, 1 *μ*M), and vasorelaxation to both anandamide and NADA was inhibited by PPAR*γ* antagonism (GW9662, 1 *μ*M). Pharmacological inhibition of *de novo* protein synthesis, nitric oxide synthase, and super oxide dismutase abolished the responses to anandamide and NADA. Removal of the endothelium partly inhibited the vasorelaxant responses to anandamide and NADA. Inhibition of fatty acid amide hydrolase (URB597, 1 *μ*M) inhibited the vasorelaxant response to NADA, but not anandamide. These data indicate that endocannabinoids cause time-dependent, PPAR*γ*-mediated vasorelaxation. Activation of PPAR*γ* in the vasculature may represent a novel mechanism by which endocannabinoids are involved in vascular regulation.

## 1. Introduction

Peroxisome proliferator-activated receptors (PPARs) are nuclear receptors which control the transcription of many families of genes. They have a large ligand binding pocket and are pharmacologically promiscuous, being activated by a number of structurally diverse natural and synthetic ligands including some angiotensin II receptor antagonists [1], statins [2], retinoic receptor antagonists [3], flavinoids [4], and citrus fruit compounds [5]. An increasing body of evidence now also suggests that cannabinoids activate PPARs, and this may mediate some of the biological effects of cannabinoids [6], in addition to activation of two well-established 7-transmembrane cannabinoid receptors (CB_1_ and CB_2_).

The first evidence of cannabinoid interactions with PPAR came in 2002 in a study by Kozak and colleagues who showed that lipoxygenase metabolism of the endocannabinoid, 2-arachidonoylglycerol (2-AG), produced a metabolite that increases the transcriptional activity of PPAR*α* [7]. Fu et al. (2003) then showed that the appetite-suppressing and weight-reducing effects of another endocannabinoid-related agent, oleoylethanolamide (OEA), were absent in PPAR*α* knock-out mice [8]. Guzmán et al. (2004) also showed that the stimulatory effect of OEA on lipolysis *in vivo* was absent in PPAR*α* knock-out mice [9]. Palmitoylethanolamide (PEA), which is structurally related to OEA, similarly activates PPAR*α* transcriptional activity, causing anti-inflammatory actions that were absent in PPAR*α* knock-out mice [10]. Other endocannabinoids that have been shown to activate PPAR*α* include noladin ether and virodhamine [11].

As well as activating PPAR*α*, it was shown in 2003 [12] that the synthetic cannabinoid, ajulemic acid (an analogue of a tetrahydrocannabinol metabolite) binds to and increases the transcriptional activity of PPAR*γ*. We have since shown that the principal active ingredient of *Cannabis sativa*, Δ^9^-tetrahydrocannabinol (THC), activates the transcriptional activity of PPAR*γ* and stimulates adipogenesis, a PPAR*γ* property [13]. The endocannabinoids anandamide and 2-AG have anti-inflammatory effects which are sensitive to PPAR*γ* antagonism [14, 15], although it was not clear whether these effects were through activation of PPAR*γ* directly, or via metabolites of the endocannabinoids. Subsequent research has shown that anandamide directly binds to PPAR*γ* [16, 17], activates PPAR*γ* transcriptional activity, and stimulates the differentiation of fibroblasts to adipocytes [16]. Other cannabinoids that activate the transcriptional activity of PPAR*γ* include the endocannabinoid/endovanilloid, N-arachidonoyl-dopamine (NADA), the synthetic cannabinoids WIN55212-2 and CP55940, and the phytocannabinoid, cannabidiol [18].

We have shown that THC causes time-dependent, endothelium-dependent, PPAR*γ*-mediated vasorelaxation of the rat isolated aorta [13]. This response was dependent on nitric oxide (NO) and superoxide dismutase (SOD) activity [13]. Furthermore, subsequent studies showed that 2-hour incubation with THC (10 *μ*M) *in vitro* blunts subsequent contractile responses and enhances vasodilator responses in isolated arteries, which was also inhibited by a PPAR*γ* antagonist [19]. These experiments similarly indicated a role for increased SOD activity stimulated by THC. Together, these studies suggest that THC, through activation of PPAR*γ*, leads to increased synthesis of SOD, promoting vasorelaxation by preventing NO being scavenged by endogenous superoxides. This is in agreement with research showing that, in addition to direct effects on NO production, PPAR*γ* ligands enhance NO bioavailability in blood vessels through induction of SOD [20].

There has been much interest surrounding the vascular actions of endocannabinoids. The mechanisms underpinning the acute vasorelaxant response to endocannabinoids include activation of sensory nerves [21–23], activation of the CB_1_ receptor, and activation of a novel endothelial cannabinoid receptor [23–25]. In light of the growing evidence that endocannabinoids activate PPAR*γ* [14–18], the aim of the present study was to investigate whether similar time-dependent, PPAR*γ*-mediated vasorelaxation to endocannabinoids occurs in the rat aorta as observed for THC, and to investigate the underlying mechanisms.

## 2. Material and Methods

### 2.1. In Vitro Vascular Studies

Male Wistar rats (250–350 g) were stunned by a blow to the back of the head and killed by cervical dislocation. The aortae were removed rapidly and placed into cold modified Krebs-Henseleit buffer (composition, mM: NaCl 118, KCl 4.7, MgSO_4_ 1.2, KH_2_PO_4_ 1.2, NaHCO_3_ 25, CaCl_2_ 2, and D-glucose 10). The aortae were dissected free of adherent connective and adipose tissue and cut into rings 3-4 mm long, and mounted on fixed segment support pins using the Multimyograph system (Model 610M, Danish Myo Technology, Denmark) as previously described [13, 19, 23]. Once mounted, all vessels were kept at 37°C in modified Krebs-Henseleit buffer and gassed with 5% CO_2_ in O_2_. The aortae were stretched to an optimal passive tension of 9.8 mN tension. Vessels were allowed to equilibrate and the contractile integrity of each was tested by its ability to contract to 60 mM KCl by at least 4.9 mN. Vessels were contracted with a combination of U46619 (10–100 nM, a thromboxane prostanoid receptor agonist), and the *α*-adrenoceptor agonist methoxamine (1–5 *μ*M) to increase tension.

When stable contraction was maintained, the vasorelaxant effect of a single concentration of endocannabinoid or vehicle control (0.1% ethanol) on induced tone was assessed as the reduction in tone over time. The endocannabinoids chosen were anandamide (5 *μ*M) and NADA (10 *μ*M), both previously demonstrated to be PPAR*γ* ligands [14, 16–18], and PEA (10 *μ*M), which activates PPAR*α* but not PPAR*γ* [10]. For every experimental protocol, vehicle-treated and endocannabinoid-treated experiments were performed in adjacent segments of the same artery.

To assess any possible contribution of vasorelaxation mediated through cannabinoid receptors, some experiments were performed in the presence of the cannabinoid CB_1_ receptor antagonist AM251 (1 *μ*M), or the CB_2_ receptor antagonist AM630 (1 *μ*M), both added 10 minutes before contracting the vessels.

To assess the contribution of PPAR*γ* activation, some experiments were performed in the presence of the PPAR*γ* antagonist GW9662 (1 *μ*M) added 10 minutes prior to precontraction. To establish whether the time-dependent vasorelaxant effects of endocannabinoids were dependent upon *de novo* protein synthesis, some experiments were performed in the presence of the protein synthesis inhibitor cycloheximide (10 *μ*M).

To investigate the role of endothelium-derived relaxing factors in the time-dependent vasorelaxation to endocannabinoids, some vessels were denuded of their endothelium by abrasion with a human hair. The role of endothelium-derived nitric oxide (NO) was investigated using the NO synthase inhibitor N^G^-nitro-L-arginine methyl ester (L-NAME, 300 *μ*M, present throughout). To establish whether endocannabinoids cause increased expression of superoxide dismutase (SOD) activity, some experiments were performed in the presence of the SOD inhibitor diethyldithiocarbamate (DETCA, 3 mM), added 30 minutes prior to precontraction of arteries.

To assess whether the actions of endocannabinoids are due to their breakdown to other biologically active compounds that may act at PPAR*γ*, some vessels were treated with the FAAH inhibitor, URB597 (1 *μ*M, added 10 minutes prior to precontraction).

### 2.2. Statistical Analysis

In each protocol, the number of animals in each group is represented by *n*, and values are expressed as mean ± SEM. The difference between endocannabinoid-treated and vehicle-treated vessels (adjacent segments from the same aorta) under each experimental protocol were analysed by paired Student's *t*-test.

### 2.3. Drugs

All drugs were supplied by Sigma Chemical Co. (UK) except where stated. Anandamide, NADA, PEA, AM251, AM630, and GW9662 were obtained from Tocris (UK). L-NAME, DETCA, and cycloheximide were dissolved in the Krebs-Henseleit solution. Anandamide, NADA, PEA, and URB597 were dissolved in ethanol at 10 mM with further dilutions made in distilled water. AM251, AM630, and GW9662 were dissolved in DMSO to 10 mM, with further dilutions in distilled water.

## 3. Results

### 3.1. Time-Dependent Vasorelaxant Effects of Endocannabinoids

**Figure 1:**
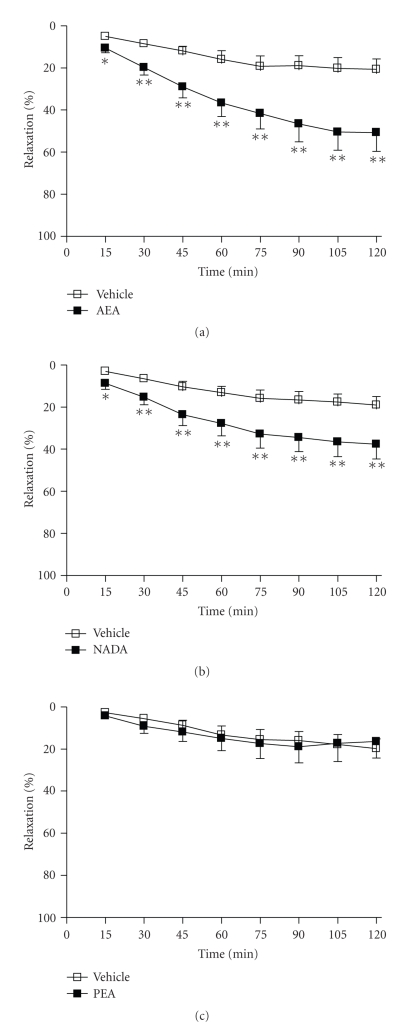
The mean vasorelaxant response to (a) AEA, (b) NADA, and (c) PEA versus vehicle (0.1% EtOH) over 2 hours in preconstricted aortae. Data are given as means with error bars representing SEM. (*P < .05, **P < .01, Student's *t*-test, *n* = 12).

Anandamide (5 *μ*M) caused significant time-dependent relaxation of the rat aorta compared to vehicle-treated arteries at all time-points over the course of 2 hours (2 hours, vehicle 21 ± 5% versus AEA 51 ± 8% relaxation, *n* = 12, *P* < .01, see Figure 1(a)). NADA (10 *μ*M) also caused significant time-dependent relaxation of the rat aorta compared to vehicle control at all time-points studied over the course of 2 hours (2 hours, vehicle 19 ± 4% versus NADA 38 ± 7% relaxation, *n* = 12, *P* < .01, see Figure 1(b)). By contrast, PEA (10 *μ*M) did not have any significant effect on the rat aorta compared to vehicle (2 hours, vehicle 20 ± 5% versus PEA 17 ± 9% relaxation, *n* = 12, Figure 1(c)).

### 3.2. Receptor Sites of Action

**Figure 2:**
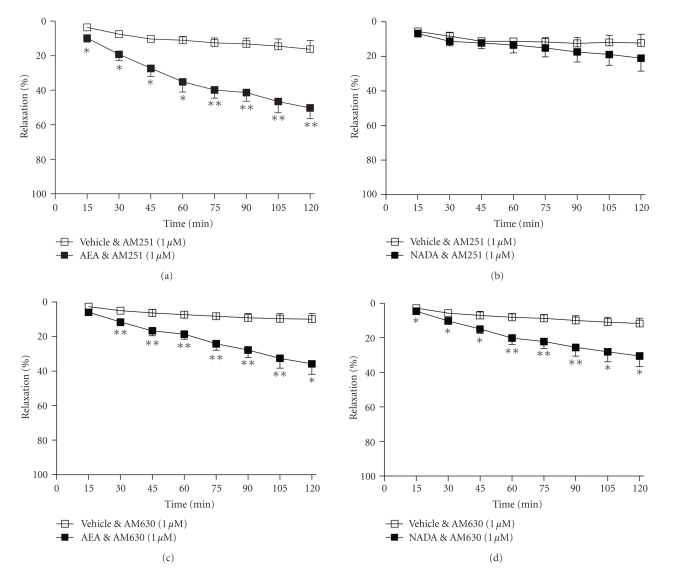
The effects of the CB_1_ receptor antagonist AM251 (1 *μ*M, (a), and (b)) and the CB_2_ receptor antagonist AM630 (1 *μ*M, (c), and (d)) on vasorelaxation to anandamide and NADA. Data are given as means with error bars representing SEM. (*P < .05, **P < .01, Student's *t*-test.)

**Figure 3:**
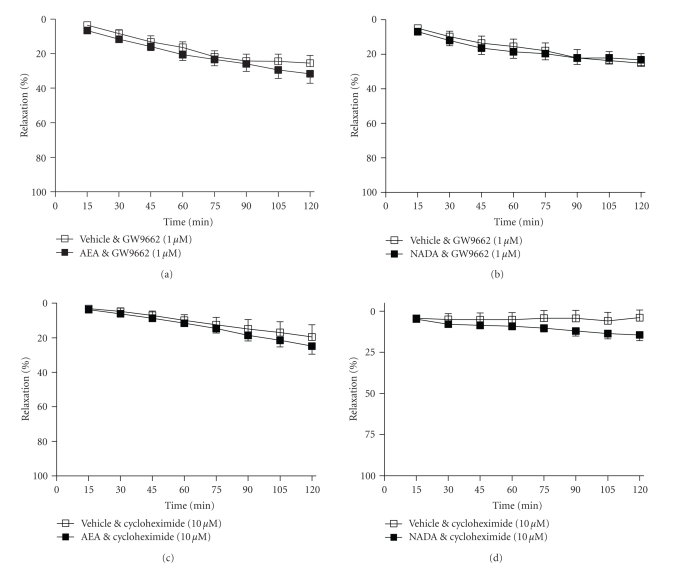
The effects of the PPAR*γ* antagonist GW9662 (1 *μ*M, (a), and (b)) and the protein synthesis inhibitor, cycloheximide (10 *μ*M, (c), and (d)) on vasorelaxation to anandamide and NADA. Data are given as means with error bars representing SEM.

In the presence of the cannabinoid CB_1_ receptor antagonist, AM251 (1 *μ*M), the vasorelaxant response to anandamide was not affected (2 hours, vehicle 16 ± 4% versus AEA 50 ± 5% relaxation, *n* = 9, *P* < .01, Figure 2(a)). By contrast, in the presence of AM251, the vasorelaxant response to NADA was abolished (2 hours, vehicle 12 ± 4% versus NADA 21 ± 6% relaxation, *n* = 9, nonsignificant, Figure 2(b)). The CB_2_ receptor antagonist AM630 (1 *μ*M) did not affect the vasorelaxant response to either anandamide (2 hours, vehicle 10 ± 3% versus AEA 36 ± 5% relaxation, *n* = 9, *P* < .05, Figure 2(c)) or NADA (2 hours, vehicle 12 ± 2% versus NADA 31 ± 5% relaxation, *n* = 8, *P* < .05, Figure 2(d)). In the presence of the PPAR*γ* receptor antagonist GW9662 (1 *μ*M), the vasorelaxant effects of both anandamide (2 hours, vehicle 26 ± 4% versus AEA 32 ± 5% relaxation, *n* = 12, nonsignificant, Figure 3(a)) and NADA (2 hours, vehicle 25 ± 4% versus NADA 23 ± 3% relaxation, *n* = 9, nonsignificant, Figure 3(b)) were abolished.

### 3.3. Mechanisms of Action

In the presence of the protein synthesis inhibitor, cycloheximide (10 *μ*M), the vasorelaxant effects of both anandamide (2 hours, vehicle 20 ± 6% versus AEA 25 ± 4% relaxation, *n* = 8, nonsignificant, Figure 3(c)) and NADA (2 hours, vehicle 4 ± 4% versus NADA 14 ± 3% relaxation, *n* = 9, nonsignificant, Figure 3(b)) were abolished.

**Figure 4:**
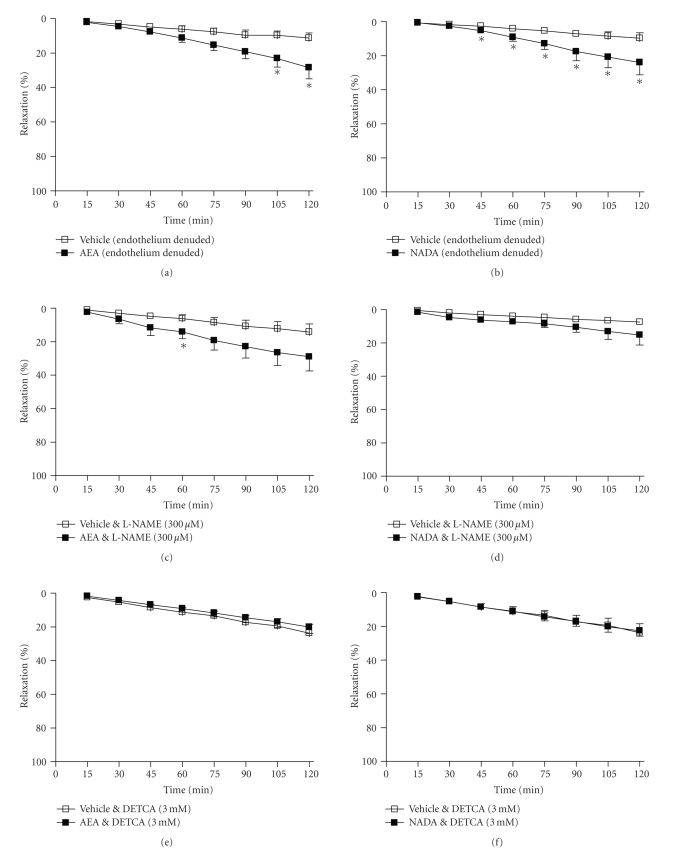
The effects of removing the endothelium ((a), (b)), inhibiting nitric oxide synthase (L-NAME, 300 *μ*M, (c), and (d)), and inhibiting superoxide dismutase (DETCA, 3 mM, (e), and (f)) on vasorelaxation to anandamide and NADA. Data are given as means with error bars representing SEM. (*P < .05, Student's *t*-test).

Removal of the endothelium limited the vasorelaxant effects of anandamide such that arteries treated with anandamide were significantly different from vehicle-treated arteries only at 105 and 120 minutes (2 hours, vehicle 11 ± 3% versus AEA 29 ± 6% relaxation, *n* = 11, *P* < .05, see Figure 4(a)). Similarly, removal of the endothelium limited the vasorelaxant response to NADA (2 hours, vehicle 10 ± 3% versus AEA 24 ± 6% relaxation, *n* = 9, *P* < .05, see Figure 4(b)). The NOS inhibitor, L-NAME (300 *μ*M), inhibited the vasorelaxant response to anandamide (2 hours, vehicle 16 ± 5% versus AEA 31 ± 8% relaxation, *n* = 11, nonsignificant, Figure 4(c)) and NADA (2 hours, vehicle 6 ± 1% versus NADA 15 ± 5% relaxation, *n* = 8, nonsignificant, Figure 4(c)). Similarly, the SOD inhibitor, DETCA (3 mM) abolished the vasorelaxant response to both anandamide (2 hours, vehicle 20 ± 4% versus AEA 20 ± 8% relaxation, *n* = 8, nonsignificant, Figure 4(e)) and NADA (2 hours, vehicle 24 ± 4% versus NADA 22 ± 3% relaxation, *n* = 8, nonsignificant, Figure 4(f)).

### 3.4. Endocannabinoid Metabolism

**Figure 5:**
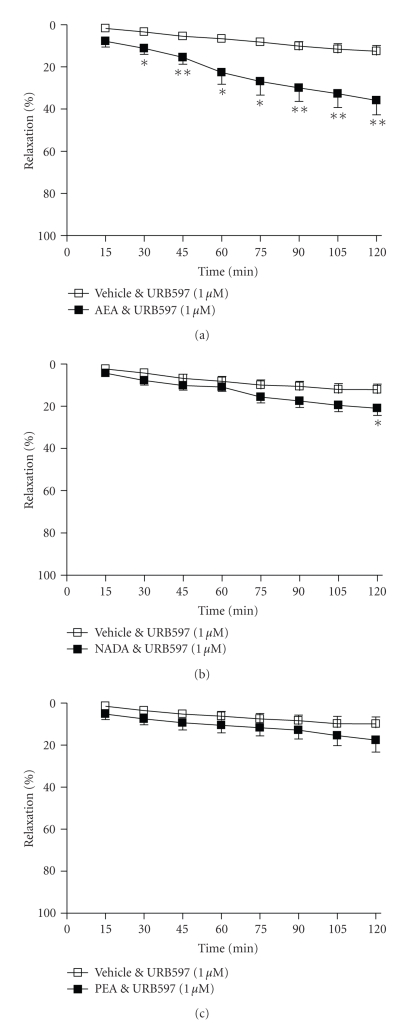
The effects of the FAAH inhibitor, URB597 (1 *μ*M) on vasorelaxation to (a) anandamide, (b) NADA, and (c) PEA. Data are given as means with error bars representing SEM. (*P < .05, **P < .01, Student's *t*-test).

The presence of the FAAH inhibitor, URB597 (1 *μ*M) did not affect the vasorelaxant effect of anandamide (2 hours, vehicle 13 ± 2% versus AEA 36 ± 6% relaxation, *n* = 10, *P* < .01, Figure 5(a)), and did not alter the vascular response to PEA (2 hours, vehicle 10 ± 2% versus PEA 18 ± 4% relaxation, *n* = 7, nonsignificant, Figure 5(c)). URB597 did inhibit the vasorelaxant response to NADA such that NADA-treated and vehicle-treated arteries were significantly different at 2 hours only (2 hours, vehicle 12 ± 2% versus NADA 21 ± 3% relaxation, *n* = 9, *P* < .05, Figure 5(b)).

## 4. Discussion

In the present study, we have examined whether endocannabinoids cause time-dependent, PPAR*γ*-mediated vascular effects as previously shown for the phytocannabinoid, THC [13, 19]. In these studies, we demonstrate for the first time that the endocannabinoids anandamide and NADA cause PPAR*γ*-mediated, time-dependent vasorelaxation of rat aortae, which is dependent on *de novo* protein synthesis, nitric oxide production and superoxide dismutase activity. These are similar mechanisms to those found to underlie the vasorelaxant effects of the PPAR*γ* agonists, rosiglitazone [26], and THC [13].

On the basis that PPAR*γ* agonists cause time-dependent vasorelaxation of isolated aortae [13, 26], and that endocannabinoids activate PPAR*γ* [14–18], we investigated whether endocannabinoids produce time-dependent vasorelaxation. The endocannabinoids chosen were anandamide and NADA, both previously demonstrated to activate PPAR*γ* [14, 16–18], and PEA, which activates PPAR*α* but not PPAR*γ* [10]. We found that, like rosiglitazone and THC, anandamide and NADA produced a slowly developing decrease in tone of precontracted aortae that was significantly greater than that seen in vehicle-treated segments of the same artery. The vascular response to anandamide and NADA was inhibited by the PPAR*γ* antagonist, GW9662, and by inhibition of *de novo* protein synthesis. In contrast, PEA did not cause vasorelaxation of the rat aorta. This is in agreement with our previous finding that the PPAR*α* ligand, bezafibrate, does not cause time-dependent vasorelaxation of isolated aortae [13]. These results demonstrate that PPAR*γ*-, but not PPAR*α*-active endocannabinoids cause time-dependent vascular effects.

Some of the vasorelaxant effects of cannabinoids are due to activation of other target sites such as the CB_1_ or CB_2_ receptor [27], and we explored whether the vasorelaxant response to endocannabinoids might be partially mediated by any of these. We found that neither the CB_1_ nor CB_2_ receptor antagonists had any significant effect on vasorelaxation to anandamide. However, vasorelaxation to NADA was inhibited by the CB_1_ receptor antagonist, AM251. It is possible, therefore, that NADA may activate cannabinoid receptors at the cell surface, initiating intracellular signalling that may lead to PPAR*γ* activation. For example, it has been shown that statins activate PPARs through activation of extracellular signal-regulated kinase (ERK)1/2 and p38 mitogen-activated protein kinase (MAPK) [28]. Both of these pathways can be activated by cannabinoid receptor activation [29, 30].

Further analysis of the time-dependent vasorelaxant effects of anandamide and NADA showed that these responses are partially endothelium-dependent and NO-dependent, as previously demonstrated for rosiglitazone and THC [13, 26]. We have also previously demonstrated that the PPAR*γ*-mediated vascular effects of cannabinoids are due to increases in SOD activity [13, 19]. Similarly, in the present study, the time-dependent effects of anandamide and NADA were abolished in the presence of a SOD inhibitor, DETCA, suggesting the vasorelaxant effects of anandamide and NADA are mediated by upregulation of SOD, preventing NO of being scavenged by endogenous superoxides. This is in agreement with other work showing PPAR*γ* ligands cause the induction of Cu/Zn-SOD [20], and with numerous studies that have shown that PPAR*γ* ligands increase NO production and bioavailability *in vitro* and *in vivo* [31–34].

There are several potential mechanisms by which cannabinoids can activate PPAR*γ* including direct binding, metabolism to other compounds that activate PPARs, or via intracellular signalling cascades. To establish whether endocannabinoids are metabolised into PPAR*γ*-active compounds, we performed some experiments in the presence of the FAAH inhibitor, URB597. The vasorelaxant effects of anandamide were not affected by URB597, which is consistent with previous studies showing that anandamide directly binds to PPAR*γ* [16, 17]. It also suggests that prolonging the effects of anandamide by preventing its breakdown does not enhance the PPAR*γ*-mediated vasorelaxant response. By contrast, the vasorelaxant effects of NADA were inhibited by URB597, suggesting that it is the conversion of this compound to PPAR*γ*-active metabolites that mediate the effects of NADA. There are no data presently available demonstrating a direct interaction between NADA and the PPAR*γ* ligand binding domain.

In summary, these data provide evidence for the first time that the endocannabinoids anandamide and NADA, but not the related acylethanolamide PEA, activate PPAR*γ* in the vasculature, leading to NO-dependent vasorelaxation. PPAR*γ* agonists have a number of positive cardiovascular effects, which include increased availability of NO, *in vivo* reductions in blood pressure and attenuation of atherosclerosis [35–37]. Similarly, endocannabinoids have a number of beneficial effects on the cardiovascular system such as cardiac protection [38–40], benefits in hypertension [41, 42], and potential benefits in atherosclerosis [43]. PPAR*γ* activation by some endocannabinoids may represent a novel mechanism by which they are involved in the regulation of the cardiovascular system.
